# Role of cerebroventricular size and surgical placement in modulating catheter flow distribution

**DOI:** 10.1186/s12987-026-00786-6

**Published:** 2026-03-17

**Authors:** Christopher W. Roberts, Brandon G. Rocque, Leopold Arko IV, Neena I. Marupudi, Sandeep Sood, Elise Yoon, Elliot Widd, Bryn A. Martin, Carolyn A. Harris

**Affiliations:** 1 Department of Chemical Engineering and Material Science, Wayne State University, 5050 Anthony Wayne Dr, Detroit, MI 48202 USA; 2 Division of Pediatric Neurosurgery, Department of Neurosurgery, University of Alabama at Birmingham, Children’s of Alabama, Birmingham, AL USA; 3 Departments of Neurosurgery and Pediatric Neurosurgery, Wayne State University School of Medicine and Children’s Hospital of Michigan, 3901 Beaubien Boulevard, 2nd Floor Carl’s Building, Detroit, MI 48201 USA; 4 Departments of Neurosurgery, University of Michigan, Mott Children’s Hospital, Ann Arbor, MI 48109 USA; 5 Department of Neurosurgery, Henry Ford Providence Hospital, Michigan State University College of Human Medicine, 16001 W Nine Mile Rd, Southfield, MI 48075 USA; 6 Department of Biomedical Engineering, Wayne State University, 818 W Hancock St, Detroit, MI 48202 USA; 7 Flux Neuroscience, LLC, Troy, ID 83871 USA; 8 Department of Chemical and Biological Engineering, University of Idaho, 875 Perimeter Dr, Moscow, ID 4260 USA; 9 Department of Chemical Engineering and Materials Science, Wayne State University, 6135 Woodward Avenue, Rm 3120, Detroit, MI 48202 USA

**Keywords:** Computational fluid dynamics, Hydrocephalus, Pediatric hydrocephalus, Cerebrospinal fluid, Ventricular catheter, Ventricular shunt, Ventricular morphology

## Abstract

Ventricular shunt catheters are the standard of care for managing hydrocephalus, yet 40% of implanted catheters fail within two years of implantation, mainly due to proximal catheter obstruction. Prior literature suggests that cerebrospinal fluid (CSF) preferentially enters the ventricular catheter’s holes farthest from the catheter tip, where obstruction has been thought to frequently occur. However, previous studies have relied on simplified geometries and idealized catheter positioning, limiting the ability to capture patient-specific anatomical variability. Segmental flow distribution in ventricular catheters was analyzed using ventricular models derived from pediatric patients with hydrocephalus. Two pediatric shunted patients and one pediatric externalized patient with varied sizes, ranging from enlarged (FOHR: 0.45), moderate (0.30), and small (0.29), were segmented from MRI scans and reconstructed into closed-volume 3D models. We simulated the insertion of a 4-row, 4-hole ventricular catheter using frontal, parietal, and occipital surgical approaches. A choroid plexus mimic modeled from confocal microscopy was attached to the ventricular system for inlet flow, and a constant flow rate of 0.35 mL/min was applied to simulate CSF flow inside the ventricular domain. Steady-state, laminar 3D computational fluid dynamics simulations were performed to quantify mass flow into each catheter drainage segment. Occipital placement consistently concentrated flow (> 81%) in the fourth (most distal) segment across all ventricular sizes. In contrast, frontal and parietal approaches showed variable flow distributions dependent on ventricular morphology. In the moderate ventricle, parenchymal contact obstructed distal holes, redirecting flow proximally (Segments 1–2: >99%). Segmental flow distribution was influenced by drainage hole patency, ventricular morphology, and catheter orientation. Altogether, these data suggest that anatomical or approach dependent shifts result in specific tissue contact changes and therefore change in flow distribution. A larger sample scale study is needed to understand variability resulting from heterogeneous catheter placement in varying ventricular morphologies.

**Supplementary Information:**

The online version contains supplementary material available at 10.1186/s12987-026-00786-6.

## Introduction

Hydrocephalus is a neurological condition characterized by an imbalance of production and absorption of cerebrospinal fluid (CSF) in the ventricular system. The standard treatment involves implantation of a shunt system, which diverts excess CSF from the ventricles to the peritoneal cavity with a ventricular catheter (VC) connected to a one-way valve and a distal catheter segment. Despite decades of clinical use, shunt systems continue to exhibit high failure rates, with approximately 40% failing within the first two years [1] and nearly 85% within ten years [2]. High shunt failure rates in pediatric hydrocephalus contribute to repeat hospitalizations, increased healthcare costs, and developmental challenges for affected children [3, 4]. Mechanical obstruction of the proximal catheter remains one of the most common causes of failure [1]. Obstruction often arises from tissue blocking the drainage holes, including infiltration of the ventricular wall tissue, choroid plexus tissue, blood clots, and cellular debris, which can impair CSF outflow, leading to proximal failure [5]. Prior studies have suggested that ventricular catheter obstruction may be influenced by flow dynamics within the catheter [6]. Specifically, it has been hypothesized that cerebrospinal fluid preferentially enters the hole’s farthest from the proximal catheter tip, where hydrodynamic resistance is lower, resulting in higher localized flow gradients at those sites [6, 7]. In a sample of explanted catheters from revision surgeries, tissue blockages were frequently observed in the holes farthest from the tip, while the holes closest to the tip remained patent [6]. Idealized experimental and computational models support this theory of flow distribution, showing preferential flow through holes farthest from the tip [7, 8]. 

However, the in vivo ventricular fluid environment is complex, due to anatomical variability among patients. Prior studies have not incorporated patient-specific shunted ventricular morphology or clinically relevant catheter placement, limiting their ability to investigate the relationship between flow dynamics and obstruction sites. Moreover, the influence of ventricular size and catheter location on catheter flow fields remains largely unexplored. To address these limitations, our study investigates whether ventricular morphology and catheter placement influence flow distribution within the catheter. Using segmented MRI data from two pediatric patients with shunted hydrocephalus and one pediatric externalized patient, we generated three-dimensional (3D) ventricular models and conducted computational fluid dynamics (CFD) simulations under clinical configurations. We evaluate whether ventricular anatomy and catheter location affect catheter flow fields and, by extension, may contribute to the observed patterns of catheter obstruction [5]. This approach bridges clinical imaging with mechanistic modeling to provide new insight into flow-mediated catheter failure and inform future placement strategies.

### Sample collection

Patient clinical data were collected at the Children’s Hospital of Michigan and Wayne State University. Written informed consent was obtained from all patients or their legally authorized representatives. The study received approval from the Wayne State University Institutional Review Board (IRB) before the initiation of any data collection. All MRI scans were de-identified by the Department of Radiology in accordance with IRB regulations. To evaluate the effect of ventricular morphology on VC flow dynamics, pediatric neurosurgeons selected three patients based on etiology, prior surgical history, and visual assessment of ventricular anatomy. Ventricular geometries were selected to reflect clinically representative morphologies observed in routine hydrocephalus care at the participating centers. Patient 1 was selected to represent a case of severely enlarged hydrocephalus, characterized by enlarged frontal, occipital, and temporal horns with relatively symmetric enlargement across all ventricular compartments. Patient 2 was chosen to represent a moderately enlarged, symmetric morphology, in which all ventricular components were uniformly expanded but with a smaller ventricular overall volume than in the first case. Patient 3 was selected to represent a near-slit, morphologically asymmetric configuration, with markedly narrowed frontal horns, flattened occipital horns (with right-left asymmetry), and relatively compressed temporal horns, without complete ventricular collapse.

Patient 1 is an 11-year-old male with hydrocephalus secondary to a posterior fossa pilocytic astrocytoma. He initially underwent placement of an external ventricular drain for treatment of hydrocephalus, followed by resection of the mass. Six months after tumor resection, he was found to have enlarging ventricles with transependymal flow and underwent endoscopic third ventriculostomy. Patient 2 is a 15-year-old male with hydrocephalus in association with a Chiari I malformation. He remained shunt-dependent despite multiple decompressive surgeries. He underwent multiple shunt externalizations due to abdominal pseudocysts and was eventually converted to a ventriculoatrial shunt from a ventriculoperitoneal shunt. Patient 3 is a 19-year-old female with a history of intraventricular hemorrhage of prematurity. She had a right ventriculoperitoneal shunt placed at three months of age and underwent multiple revisions due to proximal shunt failure. At age six she had placement of a lumboperitoneal shunt and ventricular reservoir for slit ventricles.

## Materials and methods

**Fig. 1:**
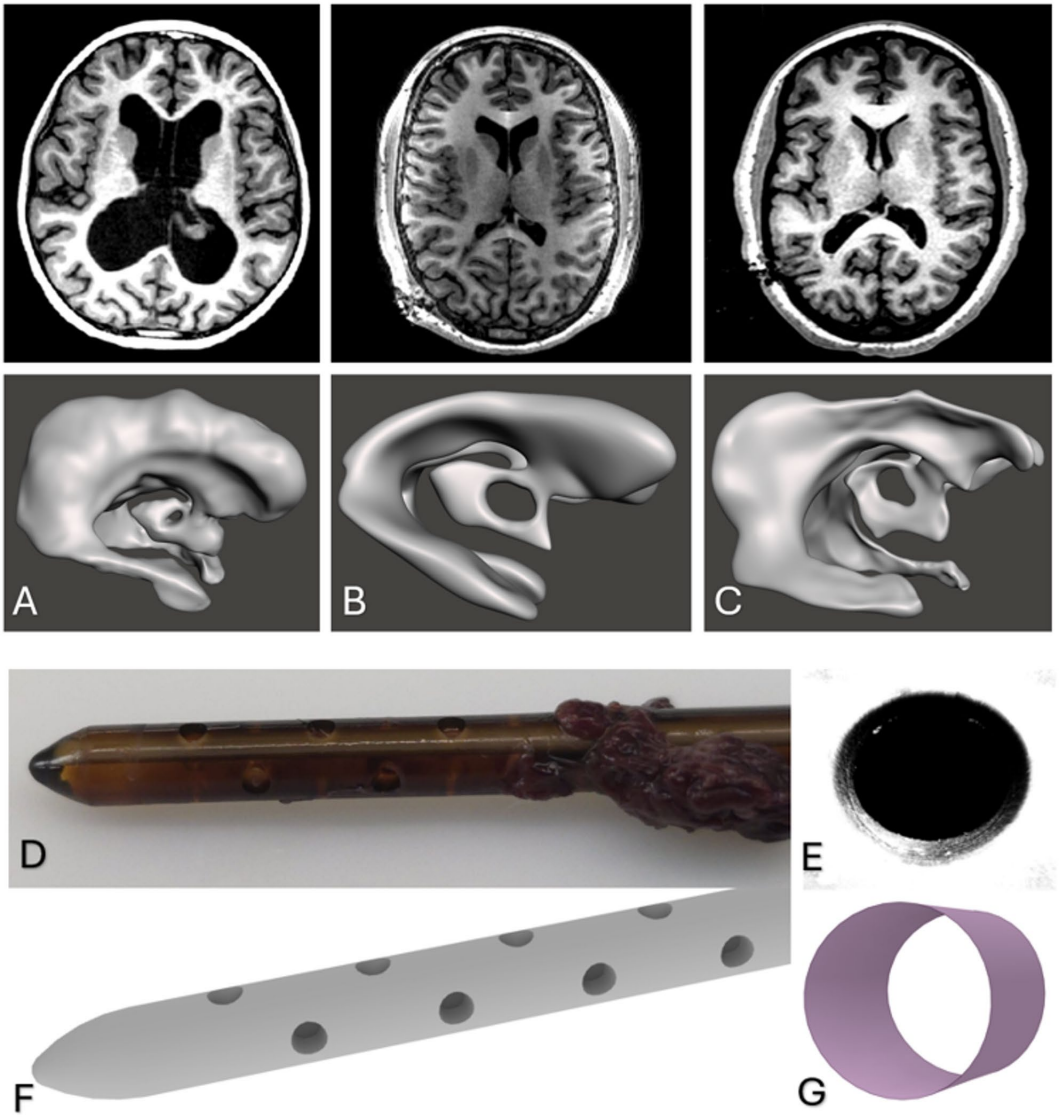
3D Segmentation Models of Enlarged Patient 1 (**A**), Moderate Patient 2 (**B**), and Small Patient 3 (**C**) Shunted pediatric ventricles, Explanted Patient Catheter (**D**), 3D Render of Explanted Catheter (**E**), Confocal Stack of Catheter Drainage Hole (**F**) STL Render of Catheter Drainage Hole in Spaceclaim (**G**)

Patient ventricles were segmented from clinical GE Medical Systems MRI scans using 3D Slicer (Fig. 1A-C) with the intent of characterizing overall ventricular size and morphology. MRI data were obtained retrospectively from routine clinical imaging protocols. Patient 1 underwent imaging on a 3.0 T GE Signa HDxt scanner using a 2D fast spoiled gradient-recalled echo (FGRE) sequence, with acquisition parameters including a slice thickness of 1.2 mm, an acquisition matrix of 256 × 128, an in-plane spatial resolution of 0.94 × 0.94 mm, and a flip angle of 30°; images were reconstructed over a 240 mm field of view. Patient 2 was imaged on a 1.5 T GE Signa HDxt scanner using a 2D FGRE sequence under a routine brain protocol without contrast, with a slice thickness of 1.2 mm, an acquisition matrix of 256 × 128, an in-plane spatial resolution of 1.17 × 1.17 mm, and a flip angle of 30°, reconstructed over a 300 mm field of view. Patient 3 underwent imaging on a 1.5 T GE SIGNA Artist scanner using a 2D single-shot fast spin echo (SSFSE) sequence, with acquisition parameters including a slice thickness of 1.4 mm, an acquisition matrix of 300 × 128, an in-plane spatial resolution of 0.55 × 0.55 mm, and a flip angle of 90°; images were reconstructed over a 280 mm field of view. DICOM segmentation [9] was performed to extract the lateral and third ventricles. The segment editor module was used to select the region of interest, followed by manual thresholding to isolate ventricular CSF from surrounding tissue.

**Fig. 2:**
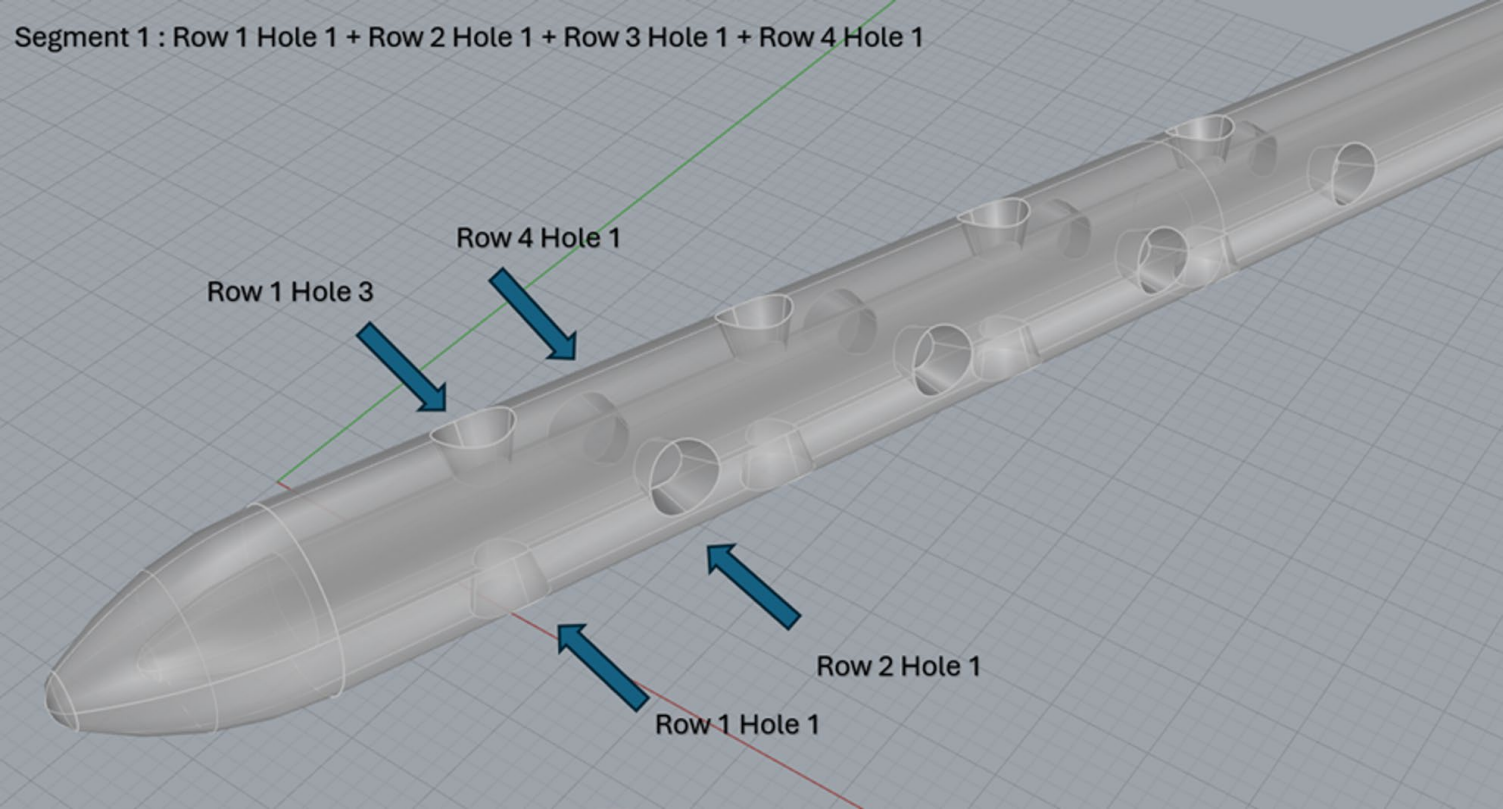
Proximal catheter drainage segments

Final ventricular segmentations were exported as STL files and refined in ANSYS SpaceClaim to correct threshold-induced surface artifacts, including intersecting surfaces, sharp edges, and non-ventricular tissue inclusion. Refinement was limited to surface cleanup and watertight closure, without altering ventricular geometry. Anatomical accuracy was verified by a pediatric neurosurgeon through direct comparison with the source MRI prior to the final model. A 4-row, 4-hole explanted ventricular catheter from our institutional biobank [5, 10] was imaged using confocal microscopy. High-resolution surface rendering was performed to reverse-engineer an accurate computer-aided design model, preserving inlet hole diameter, spacing, and internal luminal geometric curvature (Fig. 1D-G). The catheter has an inner lumen diameter of 1.4 millimeters (mm) and an outer diameter of 2.8 (mm). The drainage holes were 1.2 mm in diameter, spaced 3.7 mm apart, with the distance from the catheter tip to the center of the furthest drainage hole measuring 21.8 mm. The first hole from each row (i.e., Hole 1 from Row 1 through Row 4) was grouped and defined as Segment 1, representing the drainage region closest to the catheter tip. Similarly, the second hole from each row formed Segment 2, the third hole formed Segment 3, and the fourth hole formed Segment 4, which was furthest from the tip (Fig. 2).

**Fig. 3:**
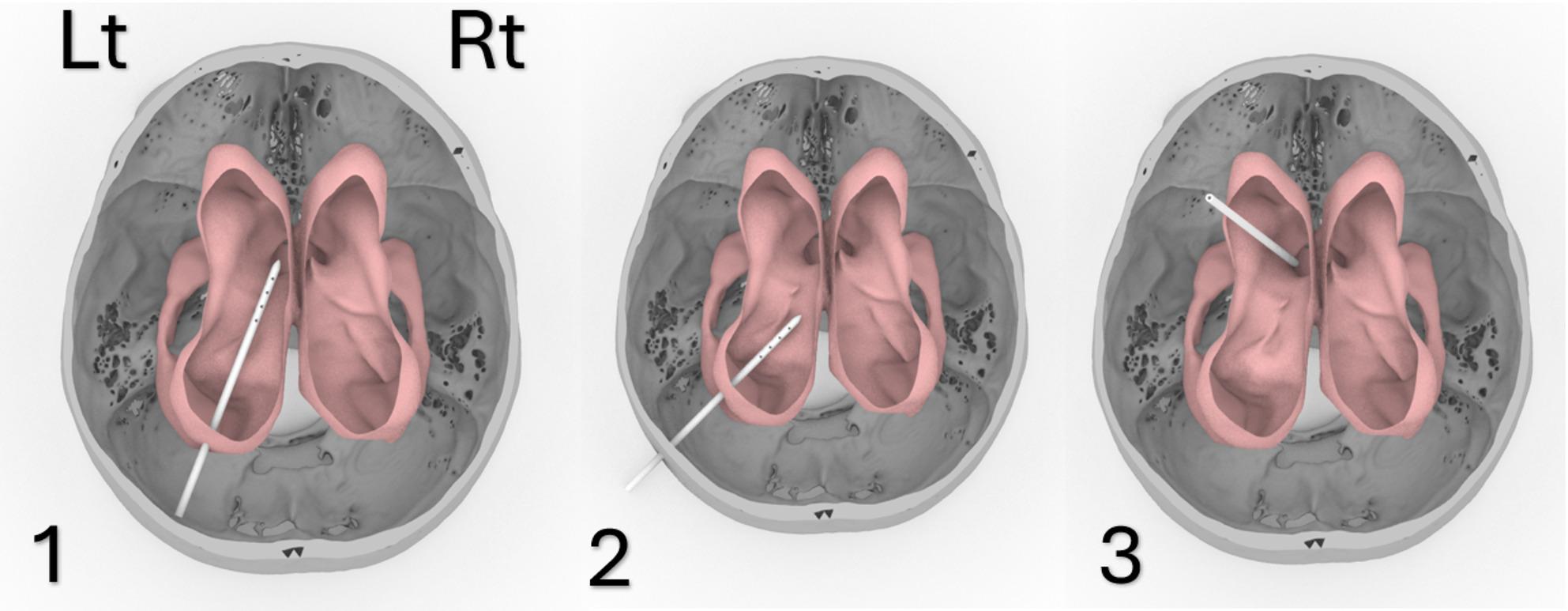
Enlarged ventricle occipital insertion (1), Parietal insertion (2), Frontal insertion

The catheter was then positioned in the three ventricular geometries representing enlarged (Patient 1), moderate (Patient 2), and small (Patient 3) hydrocephalic ventricles. In each model, the catheter was inserted using three clinical surgical trajectories: Frontal, Parietal, and Occipital [11] (Fig. 3A-C). The virtual placement was guided by anatomical landmarks derived from the segmented MRI data, and alignment was adjusted to ensure accurate insertion angles (Fig. 3, Supplemental Fig. 7–8). The catheter was consistently inserted into the left lateral ventricle in all models.

**Fig. 4:**
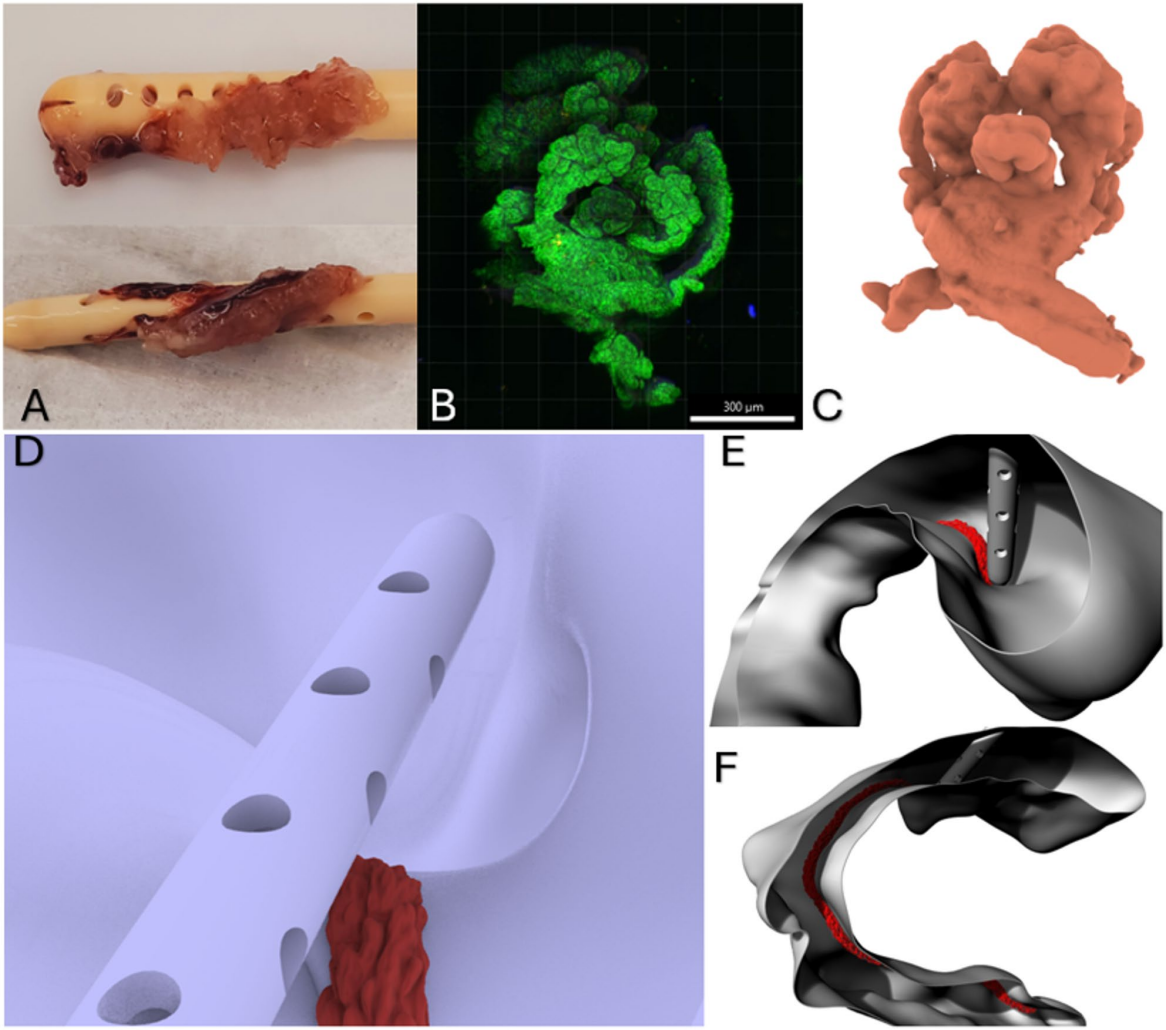
Explanted catheters with choroidal tissue (**A**), CyQUANT stained choroidal tissue (**B**), 3D render of stained of tissue (**C**), Occipital catheter placement near the foramen of monro (**D**), Representative renderings of choroid plexus withing the lateral ventricle (**E**-**F**)

To replicate physiological CSF formation [12], a custom inlet surface was developed to mimic the choroid plexus. The inflow boundary was defined based on the anatomical location of the choroid plexus, as described by Yoshida et. al [13]. Explanted patient ventricular catheters were analyzed using CyQuant labeling to visualize regions of adhered choroidal tissue [5]. High-resolution 3D stacks were acquired using a Resonance-scanning confocal microscope (RS-G4 upright microscope, Caliber ID) and processed to reconstruct surface contours (Fig. 4A-C). These contours were used to design a continuous undulating inlet geometry that spatially represented the choroid plexus. Extending from the temporal horn, through the lateral ventricles and foramen of Monro, to the roof of the third ventricle, the final inlet structure was designed to ensure uniform CSF inflow (Fig. 4D-F). The designed inlet was applied to each of the three patient-specific ventricular models, with final surface areas of 602 mm² (enlarged ventricle), 337 mm² (moderate ventricle), and 367 mm² (small ventricle), respectively, constrained by the anatomical size and shape of each ventricle.

### Computational modeling methods

**Fig. 5:**
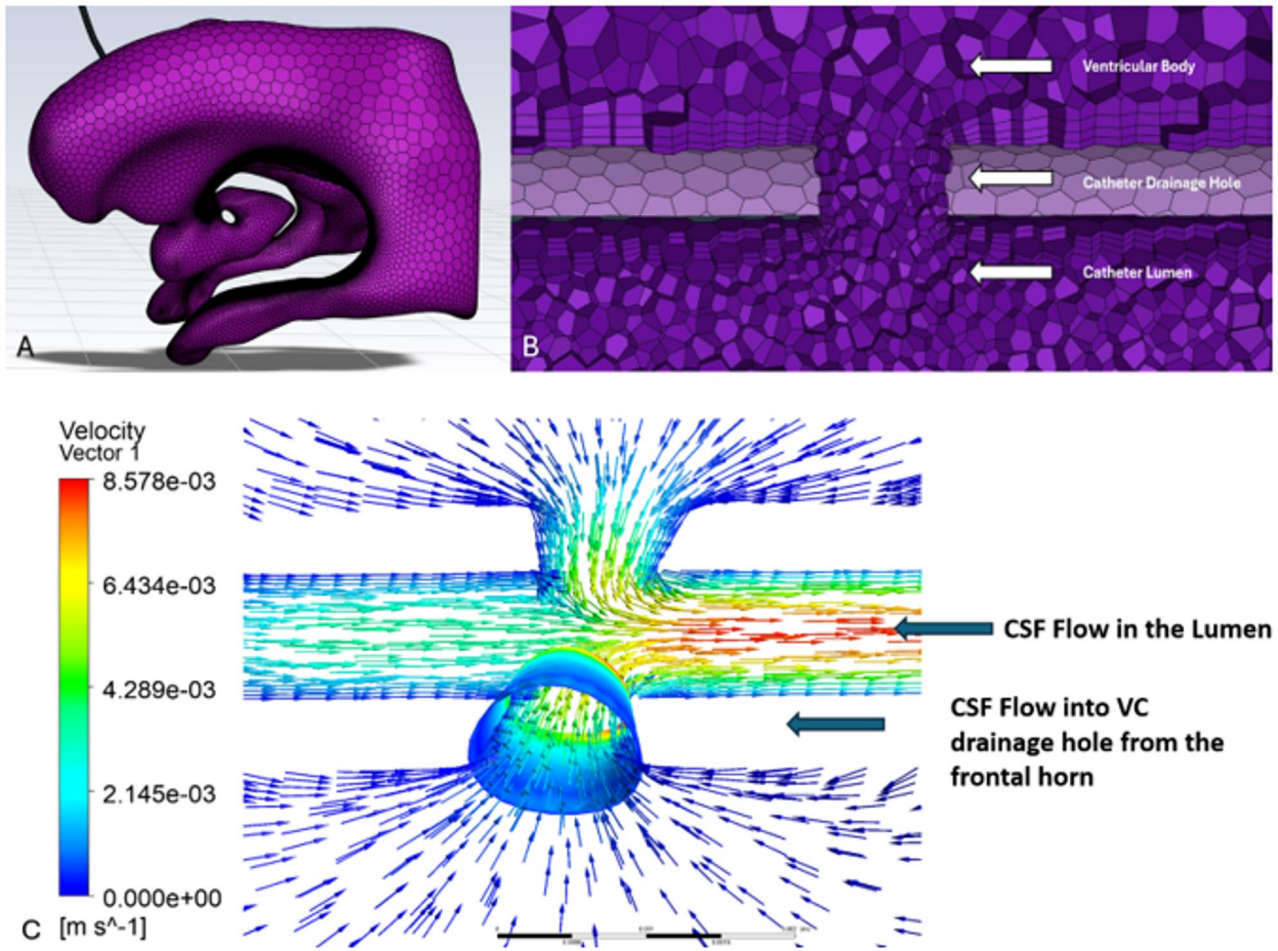
Computational surface mesh of the ventricles and catheter (**A**), Volume mesh showing element size variation within the catheter holes, lumen, and body (**B**), Visualization of CSF flow field indicating flow directionality into catheter holes (**C**)

The ventricular models were imported into ANSYS Fluent Meshing and discretized using a non-uniform, unstructured polyhedral mesh [14]. The mesh was generated using curvature and proximity-based sizing, so that smaller elements were automatically placed along the VC surfaces and in narrow gaps, while larger elements were used in the ventricular body. A uniform growth rate of 1.2 was imposed throughout the domain, and boundary-layer inflation was applied to the catheter drainage holes and luminal surfaces to resolve near-wall velocity gradients [15]. A polyhedral volume mesh was employed to enable smooth gradation from coarse ventricular elements to finer elements at the catheter (Fig. 5A-B). The ventricular surface was modeled as a rigid body [16, 17]. Cerebrospinal fluid (CSF) was treated as an incompressible, Newtonian fluid with a density of 998.3 kg/m^3^ and dynamic viscosity of 0.89 mPa⋅ s, corresponding to physiological conditions at 37 °C [18]. A constant inflow rate of 0.35 mL/min was assigned at the choroid plexus inlet surface [19–21]. A constant-pressure outlet condition was applied at the distal catheter lumen to represent valveless outflow. A coupled pressure-velocity scheme was used, with second-order discretization for pressure and second-order upwind for the continuity and momentum equations. Simulations were run under steady-state [7, 8], laminar conditions using ANSYS Fluent Solver (Canonsburg, PA, 2024 R2). Convergence residual criteria were set at 1 × 10⁻⁶.

Mesh convergence was evaluated by comparing velocity values sampled along a 1000-point rake across coarse, medium, and fine meshes. The rake was placed to capture flow from the catheter tip, across the drainage-hole segments, and into Segment 4 to compare varying flow speeds (Supplemental Fig. 5–6). As described by Khani et al., grid sensitivity can be quantified by assessing the maximum relative error (Supplemental Fig. 4) between coarse, medium, and fine meshes along such rakes [22]. Using this approach, a maximum medium-to-fine difference of 4.64% was observed, indicating that further refinement beyond the medium mesh produced only minor changes in the velocity field and that the medium mesh provided sufficient resolution for subsequent analyses. Mass flow rates were recorded at each drainage hole and at the lumen outlet to assess flow distribution (Fig. 5C).

## Results

**Fig. 6:**
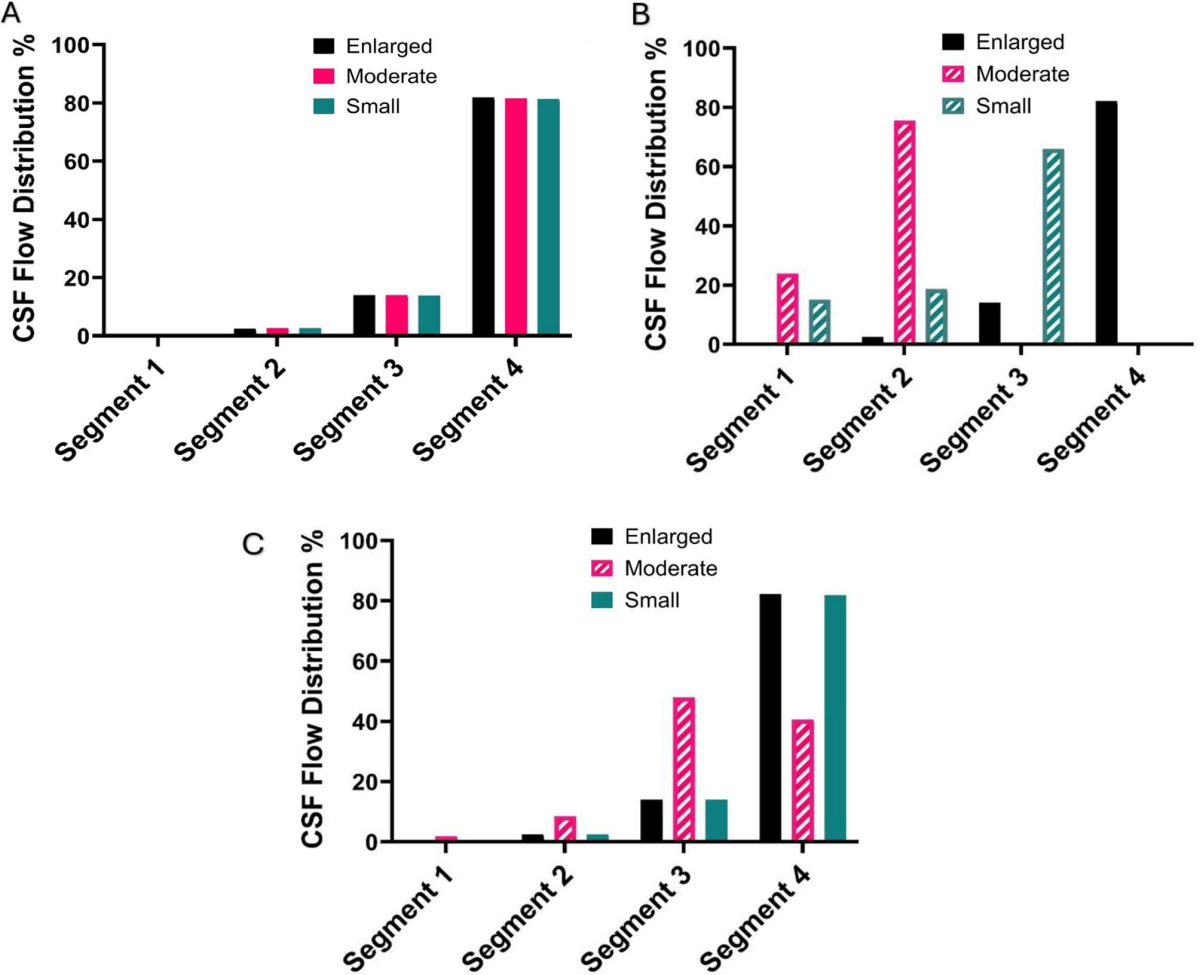
Flow distribution for occipital placement (**A**), Frontal placement (**B**), Parietal placement (**C**)

For all three ventricular volumes, occipital placement resulted in flow concentrated primarily through Segment 4, which carried 81.9%, 81.6%, and 81.4% of total inflow in the enlarged, moderate, and small ventricles, respectively. Solid bars denote segments with fully patent drainage holes, whereas cross-hatched bars indicate segments with reduced drainage due to spatial obstruction. Segment 3 contributed 13.9–14.0% across all models, while Segments 1 and 2 accounted for less than 3% combined. The overall flow distribution remained consistent across different ventricle sizes for this approach (Fig. 6A). The percentage of flow through each catheter segment is reported in Supplemental Table 1.

**Fig. 7:**
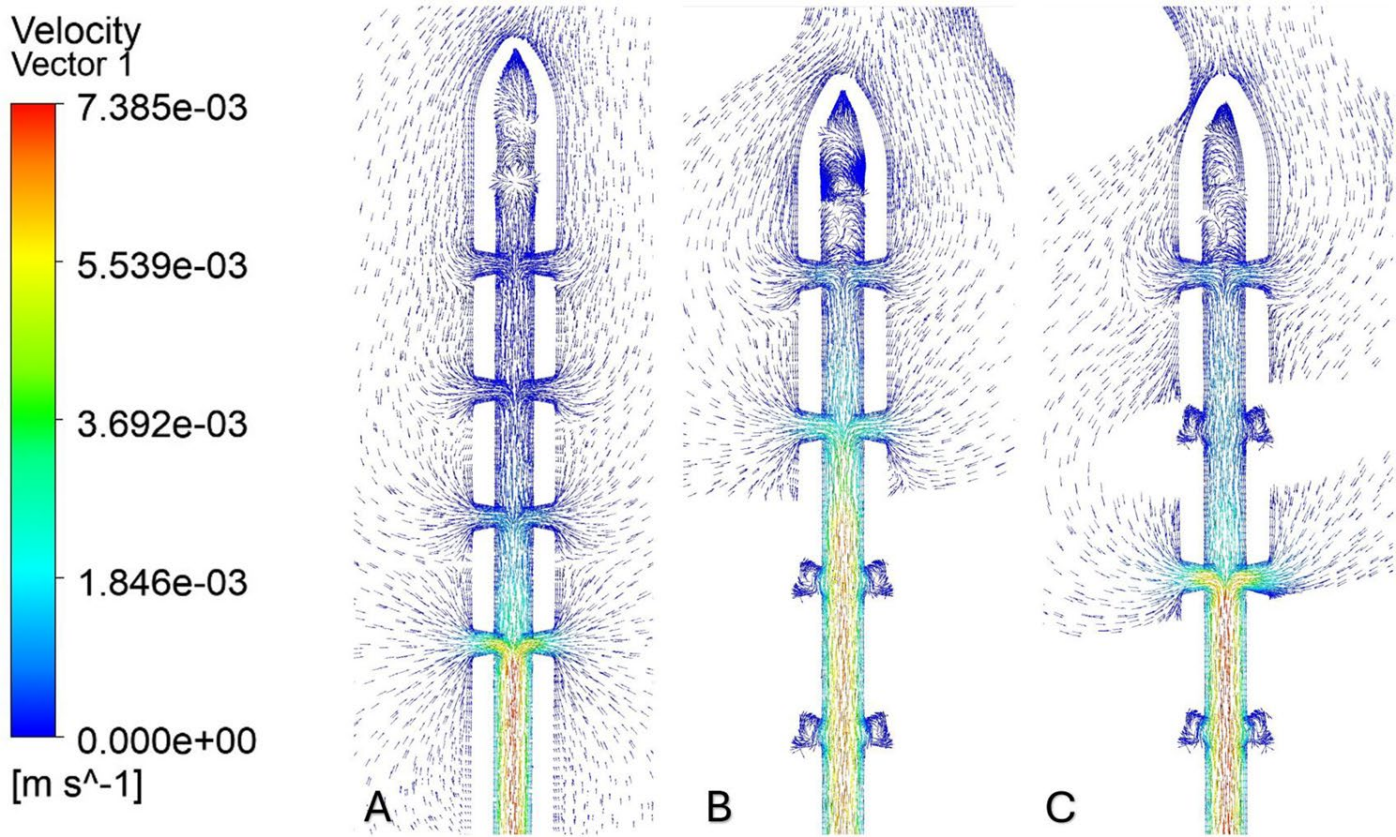
Flow distribution for frontal placement in an enlarged (**A**), Moderate (**B**), Small (**C**) Ventricle

Flow distribution in the frontal approach demonstrated variability in relation to ventricular size (Figs. 6B and 7). Segments located outside the ventricular cavity or in direct contact with the ventricular wall were considered anatomically obstructed, as such positioning restricted CSF inflow. In the enlarged ventricle, most of the flow entered through Segment 4 (82.2%), with lesser flow observed in Segments 3 (14.1%), 2 (2.5%), and 1 (0.5%). However, in the moderate ventricle, flow shifted significantly toward Segment 1 (23.9%) and 2 (75.6%), with minimal flow in Segments 3 (0.03%) and 4 (< 0.01%). In the small ventricle, Segments 1 and 2 carried 15.1% and 18.7% of the inflow, respectively, while Segment 3 exhibited the highest concentration at 66.0%. Segment 4 contributed minimally, with only 0.037% of the total flow. Flow patterns associated with the parietal approach were more gradually distributed (Fig. 6C). In the enlarged ventricle, flow remained concentrated in Segment 3 (14.0%) and Segment 4 (82.2), with Segments 1 and 2 accounting for 0.5% and 2.5%, respectively. In the moderate ventricle, the flow was distributed across Segments 4 (40.6%), 3 (47.9%), 2 (8.5%), and 1 (1.9%). For the small ventricle, Segment 4 (81.9%) accounted for the highest concentration of flow, followed by Segment 3 (14.0%), Segment 2 (2.5%), and Segment 1 (0.5%).

## Discussion

This study investigated the impact of ventricular size and surgical placement on CSF distribution through a ventricular catheter. Using pediatric hydrocephalic ventricle geometries and a standardized 4-row, 4-hole catheter, we assessed intraventricular flow distribution patterns under clinical placement strategies. Across all models, occipital placement consistently concentrated flow in Segment 4, regardless of ventricular size, despite significant variation in the spatial distance between catheter holes and adjacent ventricular walls across surgical approaches. This suggests that, for the specific catheter positions and ventricle sizes analyzed, anatomical proximity of the catheter holes with respect to the ventricular surface was not close enough to influence regional inflow patterns. Instead, flow appeared to follow a path of least hydraulic resistance within the catheter, with holes furthest from the tip preferentially capturing most of the inflow regardless of the approach-specific anatomical alignments modeled. Similar findings were reported by Galarza et al. [7] and Weisenberg et al. [8], who demonstrated that catheters placed centrally within large CSF volumes exhibited negligible influence from surrounding walls on flow distribution entry pathways. Galarza et al. [7] reported 50–75% inflow into the most distal segments of a commercial 16-hole catheter, whereas our 16-hole catheter displayed a higher distal concentration (95%). Differences in hole size and spacing suggest that catheter architecture may further influence flow distribution in unobstructed ventricular catheters and warrant future investigation [23–25].

**Fig. 8:**
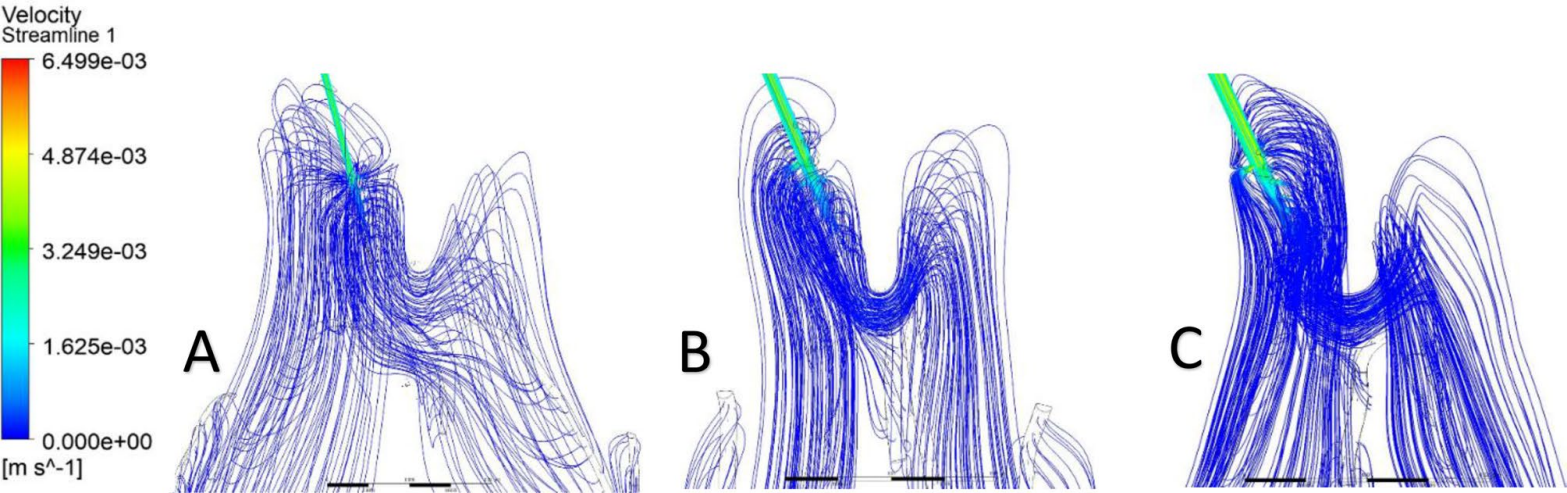
Axial flow streamlines for frontal placement (**A**) Enlarged, (**B**) Moderate, (**C**) Small

Catheter placement in the frontal approach was influenced by ventricular morphology, with flow distribution shifting significantly as morphology changed. In this placement configuration, the catheter tip was suspended just above the foramen of Monro. In this placement, flow behavior is particularly sensitive to morphology due to confined geometry and the multidirectional nature of CSF pathways in this region (Fig. 8B, C). In the enlarged ventricle, flow remained concentrated in Segments 3 and 4, accounting for 95.6% of the total inflow. However, in the moderate and small ventricles, flow was redistributed toward Segments 1–3. In the moderate model, drainage was entirely restricted to Segments 1 and 2, which together accounted for 99.5% of the total flow. This redistribution was not driven by proximity to the ventricular wall since Segments 1 and 2 in the moderate ventricle were spatially closer to the wall than in the enlarged model but instead resulted from catheter orientation within the narrowing intraventricular space. Specifically, in the moderate ventricle, Segments 3 and 4 were positioned outside the ventricular cavity in the compressed frontal horns and were therefore fully blocked from inflow. Although anatomically distal, segments positioned outside the ventricular cavity are functionally excluded from CSF inflow, shifting the effective inflow hierarchy toward intraventricular segments.

In the small ventricle, flow distribution again shifted, with Segment 3 contributing 66% of total inflow, followed by Segment 2 (18.7%) and Segment 1 (15.1%). Segment 4 was situated outside of the ventricle with no flow (0.037%). This redistribution can be attributed to the morphological differences in the frontal horn across ventricular sizes. While the moderate ventricle was compressed vertically, the small ventricle was laterally compressed, creating a broader frontal horn surface area that accommodated drainage from an additional segment. Interestingly, the small ventricle had a larger overall volume (20.6 mL) than the moderate one (12.5 mL), primarily due to expanded occipital and temporal horns. Despite this, its frontal horns were more compressed. Although volumetrically larger, the small ventricle is locally constrained in regions such as the frontal horns, while posterior regions are more expanded and accommodate a greater CSF volume. These findings suggest that ventricular volume alone is not predictive of catheter flow distribution behavior; rather, local anatomical morphology and the proximity of the catheter holes to the surface, the frontal horn in this case, exerts influence on segmental flow patterns in frontal placement.

**Fig. 9:**
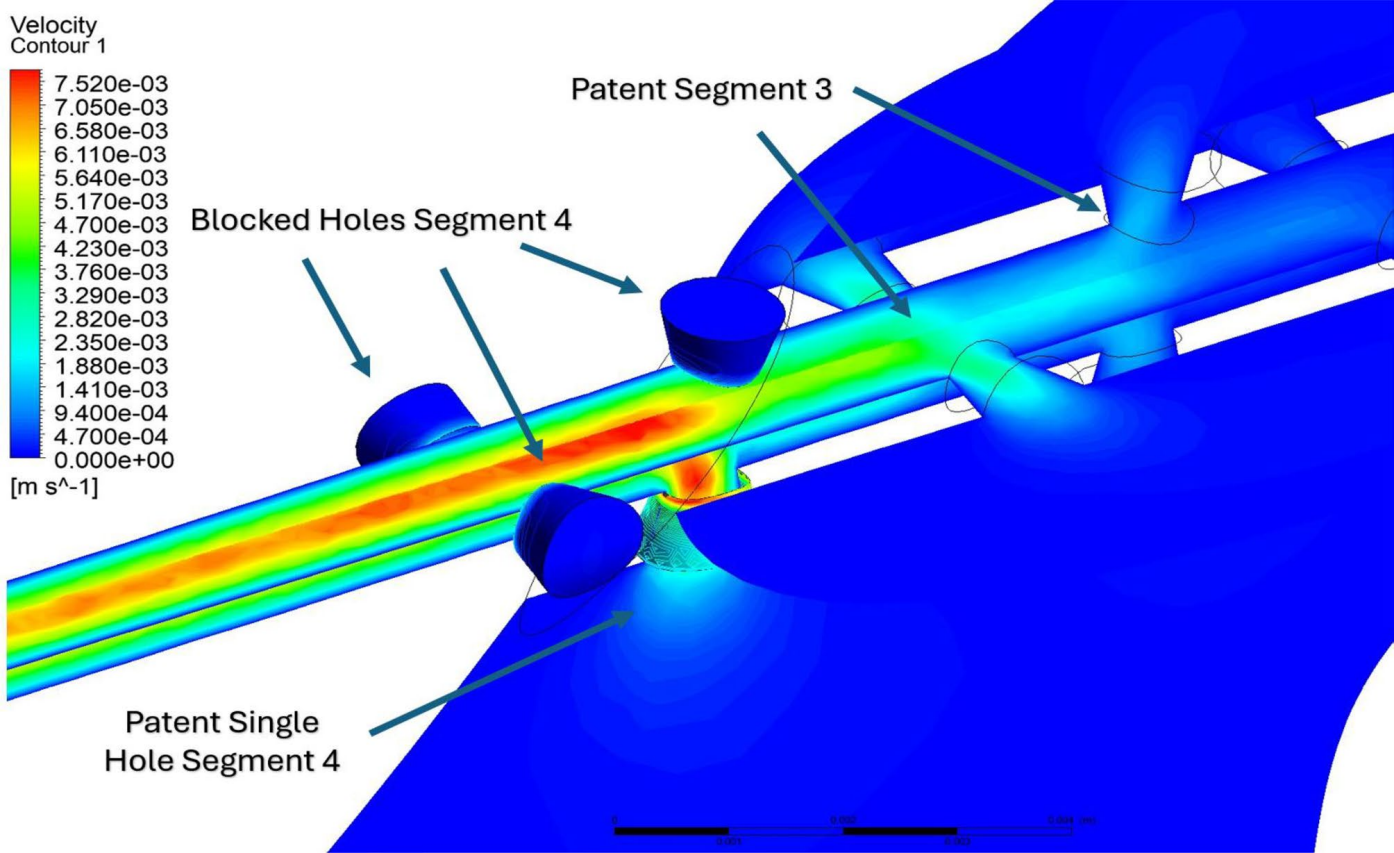
Segmental flow mapping of a catheter in a moderate ventricle (Parietal Approach)

In parietal placement, flow distribution in the enlarged and small ventricles followed a similar pattern, with Segments 3 and 4 collectively accounting for over 96% of total flow. Despite the small ventricle’s compressed frontal horns, this consistency in flow distribution remained evident. However, the small ventricle’s occipital horns provided sufficient spatial volume to accommodate all proximal drainage holes, allowing unobstructed outflow despite the overall narrowing of the ventricular system. In contrast, the moderate ventricle exhibited a more balanced distribution between Segments 3 (47.9%) and 4 (40.6%). This near-equal split is interesting given that three out of four holes in Segment 4 were obstructed by ventricular wall contact and positioned outside the ventricular cavity, whereas all four holes in Segment 3 remained patent (Fig. 9). This suggests that the number of open drainage holes does not simply govern mass flow directionality but is also shaped by local fluid pathways and the catheter’s orientation within the ventricle. While prior research [6–8] has suggested that the segment furthest from the tip typically exhibits the highest concentration of flow, our findings show that Segment 3, despite being the second most distal, displayed slightly higher flow than Segment 4 under the configuration analyzed. This indicates that local flow conditions can divert fluid preferentially into adjacent patent holes rather than concentrating entirely at the segment further from the tip of the proximal catheter.

**Fig. 10:**
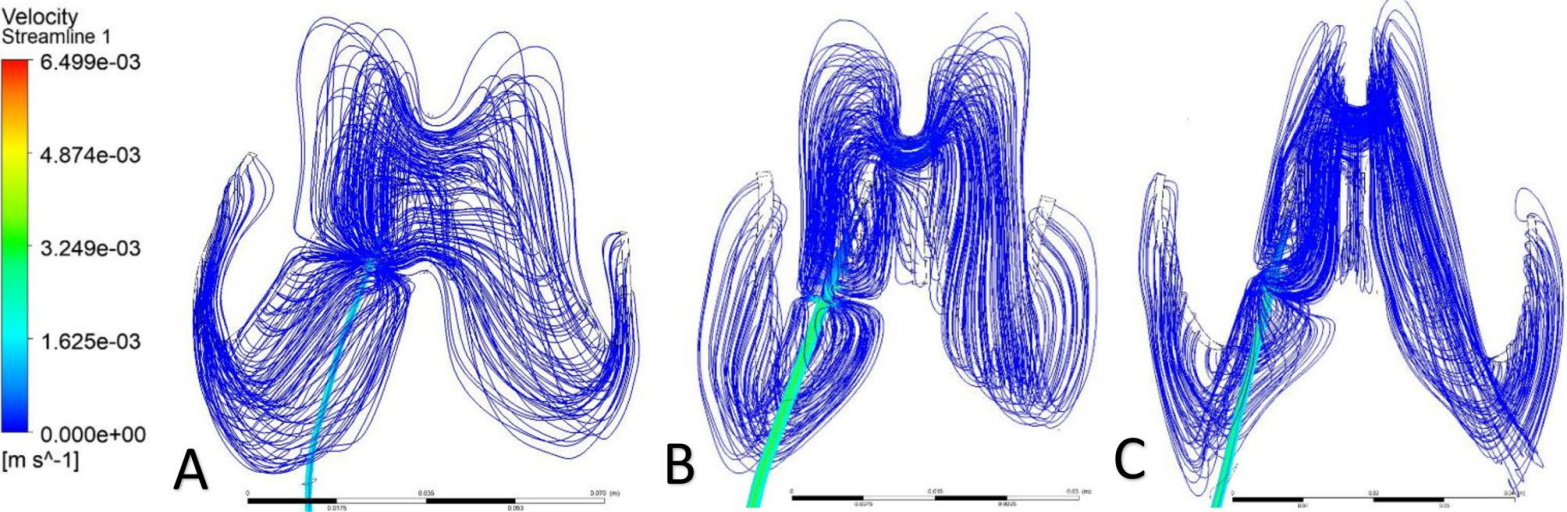
Axial flow streamlines for occipital placement (**A**) Enlarged, (**B**) Moderate, (**C**) Small

**Fig. 11:**
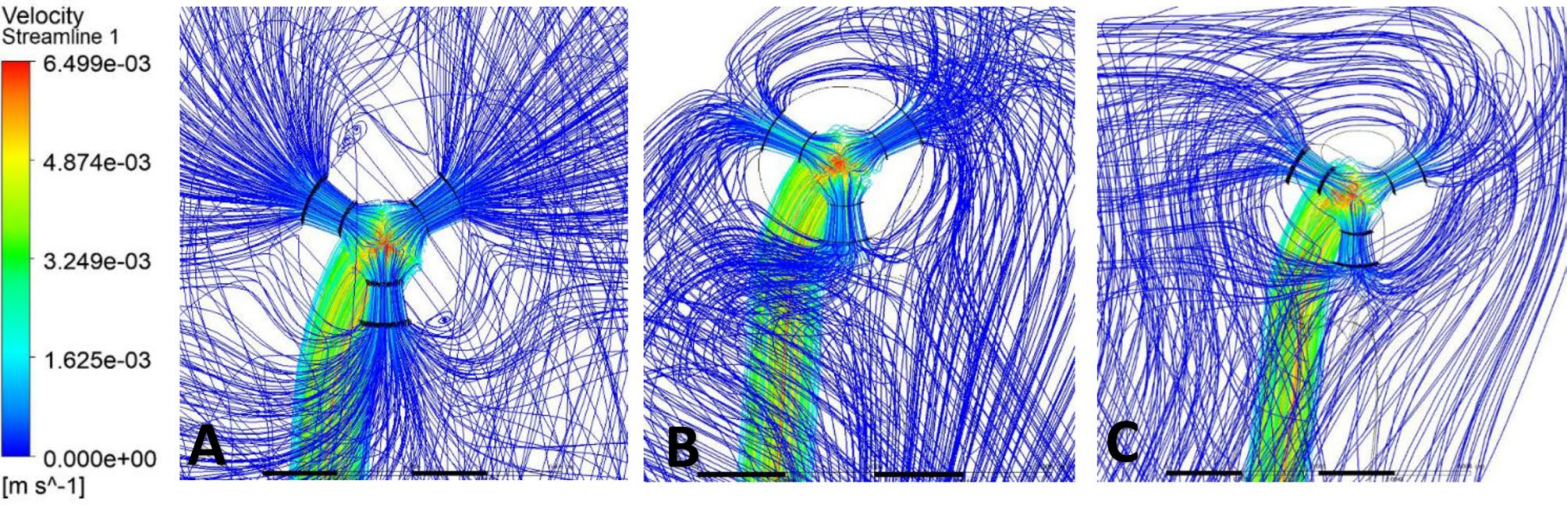
Frontal-view flow streamlines for occipital placement (**A**) Enlarged, (**B**) Moderate, (**C**) Small

**Fig. 12:**
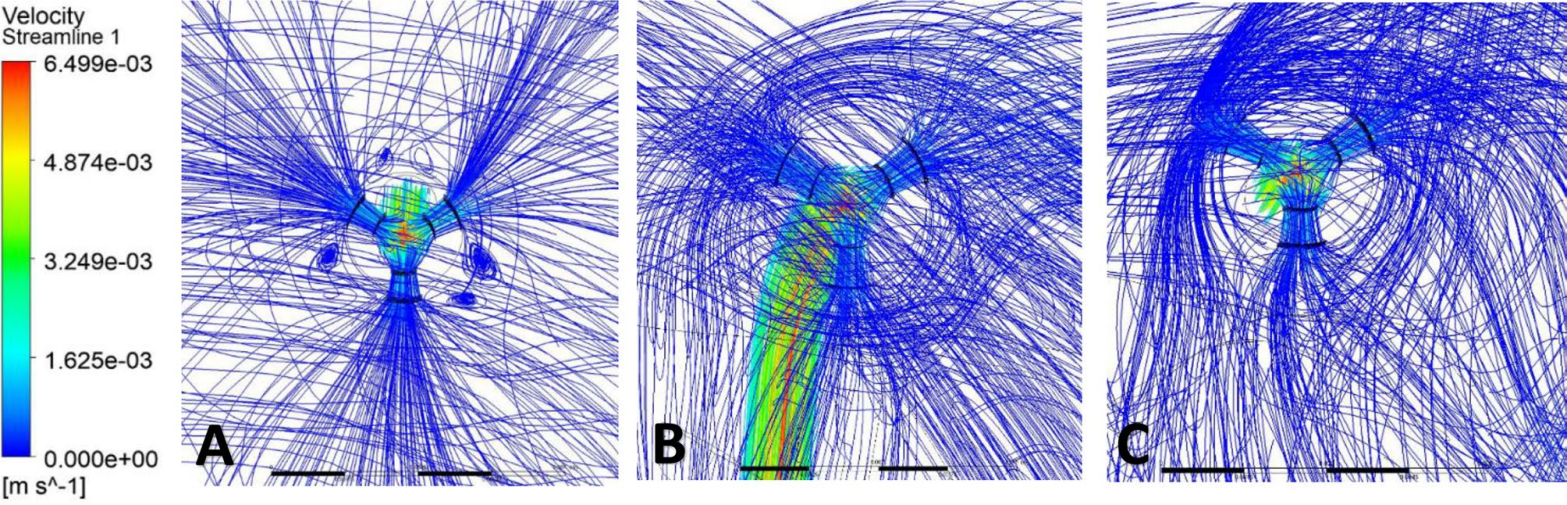
Frontal-view flow streamlines for parietal placement (**A**) Enlarged, (**B**) Moderate, (**C**) Small

**Fig. 13:**
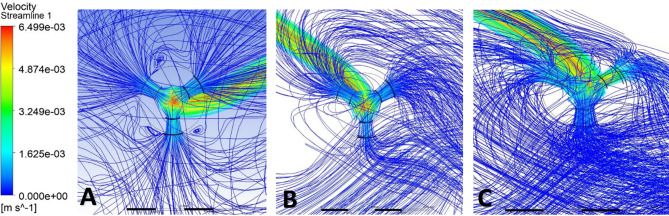
Frontal-view flow streamlines for frontal placement (**A**) Enlarged, (**B**) Moderate, (**C**) Small

Streamlines were used to visualize CSF flow pathways and assess how ventricular morphology and catheter location influence segment-level inflow. For occipital placement (Fig. 10, Supplemental Fig. 1), streamline patterns were consistent across ventricular sizes. In the enlarged ventricle, CSF entered the catheter uniformly from the surrounding ventricular space, with streamlines approaching the drainage holes in a largely perpendicular manner and distributing evenly across segments (Figs. 11A, 12A and 13A). Flow was concentrated distally, aligning with previously described catheter flow patterns [6–8]. In the moderate and small ventricles, ventricular narrowing led to increasingly compressed streamlines, particularly within the frontal horn and ventricular body. Although all drainage holes remained intraventricular, flow paths curved and wrapped around the catheter surface before entering the holes, rather than approaching directly as in the enlarged ventricle. This effect was most pronounced in the moderate and small ventricle, where streamlines closely adhered to the catheter surface prior to entry (Fig. 11B, C).

**Fig. 14:**
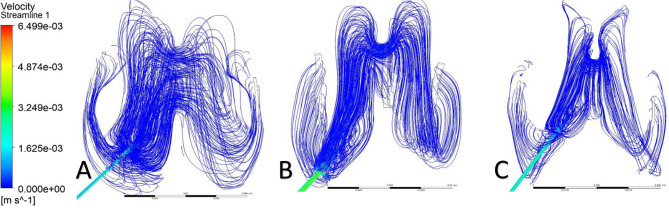
Axial flow streamlines for parietal placement (**A**) Enlarged, (**B**) Moderate, (**C**) Small

For parietal placement (Fig. 14, Supplemental Fig. 2), enlarged ventricles showed streamline behavior similar to occipital placement, with perpendicular inflow and distal concentration. In small and moderate ventricles, streamlines wrapped around the catheter (Fig. 12B, C). In the small ventricle, compressed local geometry caused streamlines to cluster near the catheter surface (Fig. 12C), with distal segments receiving flow primarily from the temporal and occipital horns (Fig. 14C). In frontal placement, the enlarged ventricle displayed streamline patterns similar to occipital and parietal approaches. For moderate frontal placement, streamlines were closely grouped in the narrowed frontal horns (Fig. 8B). Segments 3 and 4 sat outside the cavity and received no inflow, which redirected flow to Segments 1 and 2 despite some wall contact. Streamlines curved along the walls, wrapping the catheter before entering patent holes (Fig. 13B, C). In the small ventricle, Segment 3 stayed partially intraventricular, while Segment 4 was outside. Streamlines curved through the frontal horn and foramen before entering (Fig. 8C). Higher velocity was observed across several proximal segments, reflecting redistribution among partially patent holes, instead of a single distal segment (Supplemental Fig. 3C). Axial views showed streamlines more closely spaced in the small ventricle compared to the moderate ventricle, especially near the frontal horns and foramen (Fig. 8B, C). These findings show that local frontal horn morphology may expose catheter segments differently, even in ventricles of similar or larger volume, however a larger sample size is required to further confirm these results.

Localized micro-recirculation zones were observed at the catheter surface in enlarged, moderate and small ventricles (Figs. 11A, 12A and 13A). Prior radiographic studies [26] have investigated macroscopic CSF flow disturbances as imaging markers associated with ETV/CPC outcomes, the small, near-wall recirculation patterns observed here are unlikely to be detectable with current clinical imaging techniques. The potential relevance of these micro-scale flow features to CSF dynamics warrants further numerical investigation but lies beyond the scope of the present study. Holes positioned closer to the ventricular wall exhibited more tightly packed streamlines, while holes farther from the wall showed more dispersed inflow (Fig. 12C). This finding suggests that even without direct wall contact, close proximity to the ventricular surface may alter local flow convergence into the catheter. Across moderate and small ventricles, frontal views further revealed that flow entering individual drainage holes often originated from multiple intraventricular directions rather than a single dominant pathway (Fig. 11C). Streamlines converged from different regions of the ventricle before entering the same hole, highlighting the potential anatomy-driven nature of catheter inflow under constrained conditions.

Prior computational studies [6–8] have reported that the catheter segment furthest from the tip accounts for most of the CSF inflow, based on models in which distal segments remain fully within the ventricular body. Our results confirm this behavior in enlarged ventricles and in placements where distal segments remain unobstructed. However, in the moderate ventricle with parietal placement, the most distal segment contained only a single patent hole, while the adjacent proximal segment remained fully patent with multiple open drainage holes. Under these conditions, flow did not concentrate into the remaining distal hole but instead redistributed toward the segment with greater overall drainage capacity. These findings demonstrate that a patent distal segment does not necessarily receive a higher CSF inflow rate. Due to the local anatomical environment, CSF can preferentially redistribute toward upstream segments with greater effective drainage capacity, even when distal holes remain partially open. Streamline visualization in the moderate and small ventricles showed that CSF flow wrapped around the catheter and then entered the drainage segments rather than approaching the catheter segments perpendicularly. These streamline patterns demonstrate that local ventricular geometry and catheter orientation, or proximity to the ventricular wall, can actively alter streamline density and directionality. These findings demonstrate that distal position alone does not reliably predict flow concentration, as catheter flow distribution is governed by both segmental patency and anatomical location.

The ventricular system presents a complex flow environment shaped by arterial and respiratory pulsations, as well as compliant ventricular walls, all of which vary across patient populations [27, 28]. In this study, we aimed to build an idealized yet clinically motivated model that improves upon prior simplified geometries. While the ventricular geometry was based on prior patient-derived models [13, 14], we specifically used a shunted ventricle in this case to better reflect post-treatment anatomy. However, certain limitations should be acknowledged. Both the ventricle and catheter were modeled as rigid structures. Due to the low-velocity regime of intraventricular CSF flow [29–31], catheter material compliance is unlikely to influence flow distribution in the ventricular catheter.

While ventricular compliance may affect pressure wave propagation, our focus was on segmental flow directionality and distribution. Thus, wall compliance was not expected to influence these parameters within the catheter. The boundary conditions in our model assumed a continuous inflow of 0.35 mL/min, reflecting average CSF formation rates reported in the literature [19, 32–34]. To manage computational time and resources, we did not incorporate pulsatile flow in this model [31, 35]. While this approach allowed us to isolate anatomical and positional effects, future work will investigate whether physiological pulsatility impacts segmental flow distribution within anatomically variable ventricular environments. Siyahhan et al. showed that ependymal cilia contribute to near-wall CSF transport in the lateral ventricles using in vivo, patient-specific MRI-based flow reconstructions [14]. Yamada et al. utilized 4D flow MRI to characterize ventricular CSF motion and elucidate alterations in CSF dynamics associated with Hakim’s disease [13]. Fillingham et al. incorporated patient-specific phase-contrast MRI measurements and boundary conditions to account for brain tissue deformation and its influence on CSF flow [27]. Future work should explore integrating these advanced MRI-informed boundary conditions into catheter dynamics modeling to better capture patient-specific CSF dynamics in shunted populations. Lastly, the catheter was positioned centrally within the ventricle using 3D placement guidance, approximating an idealized surgical trajectory. However, in clinical practice, precise catheter positioning is challenging, especially in the absence of image guided neuroendoscopy [36]. As a result, real-world placement may lead to wall contact, even in large ventricles, which can affect local flow behavior. While we assumed an ideal placement in this study, future investigations should explore how variations in placement accuracy and wall contact affect flow distribution.

## Conclusion

In summary, this study highlights several factors that govern segmental drainage in ventricular catheters within the patient-specific cases analyzed. Ventricular volume alone does not predict CSF flow distribution. Catheter orientation and surgical placement influenced which drainage segments remained functionally inside the ventricle, specifically in the frontal horns, where local ventricular morphology affected segment patency. Proximity of drainage holes to the ventricular wall alone did not determine flow distribution: rather, the interaction between morphology and placement dictated whether segments remained patent or functionally blocked, redirecting CSF to other patent segments. Notably, the presence of a single patent hole in the segment furthest from the catheter tip did not guarantee that this region would receive the highest flow volume. Drainage segment patency does not necessarily reflect effective drainage capacity, particularly when segments are partially or fully obstructed. When distal segments were fully intraventricular and unobstructed, flow preferentially entered the furthest segments from the tip, consistent with prior models; however, under anatomically constrained conditions, flow redistributed toward segments with greater functional exposure. Overall, these findings indicate that catheter drainage patterns are shaped not only by ventricular size but also by surgical approach and local ventricular morphology. Given the limited sample size, these conclusions are restricted to the modeled cases. Future studies should incorporate larger patient cohorts, broader anatomical variability, and additional catheter placement configurations to further define how ventricular morphology and surgical placement interact to influence flow behavior. In addition, surgical placement of ventricular catheters can vary across institutions and surgeons, and even minor differences in catheter trajectory or final position may substantially alter flow patterns. These placement-dependent effects warrant further investigation.

## Supplementary Information

Below is the link to the electronic supplementary material.

Supplementary Material 1

## Data Availability

The raw datasets analyzed during the current study are not publicly available due to surrounding HIPAA privacy considerations. The deidentified clinical data and 3D processed data required to reproduce these findings are available from Dr. Carolyn Harris, Ph.D., Wayne State University, Dept. of Chemical Engineering and Materials Science, 6135 Woodward Avenue, Detroit, MI, 48202. Email: caharris@wayne.edu.

## Abbreviations

CSF: Cerebrospinal Fluid
VC: Ventricular catheter
CFD: Computational Fluid Dynamics
DICOM: Digital Imaging and Communications in Medicine
STL: Standard Tessellation Language
