# Correction: Role of cerebroventricular size and surgical placement in modulating catheter flow distribution

**DOI:** 10.1186/s12987-026-00810-9

**Published:** 2026-04-28

**Authors:** Christopher W. Roberts, Brandon G. Rocque, Leopold Arko IV, Neena I. Marupudi, Sandeep Sood, Elise Yoon, Elliot Widd, Bryn A. Martin, Carolyn A. Harris

**Affiliations:** 1 Department of Chemical Engineering and Material Science, Wayne State University, 5050 Anthony Wayne Dr, Detroit, MI 48202 USA; 2 Division of Pediatric Neurosurgery, Department of Neurosurgery, University of Alabama at Birmingham, Children’s of Alabama, Birmingham, AL USA; 3 Departments of Neurosurgery and Pediatric Neurosurgery, Wayne State University School of Medicine and Children’s Hospital of Michigan, 3901 Beaubien Boulevard, 2nd Floor Carl’s Building, Detroit, MI 48201 USA; 4 Departments of Neurosurgery, University of Michigan, Mott Children’s Hospital, Ann Arbor, MI 48109 USA; 5 Department of Neurosurgery, Henry Ford Providence Hospital, Michigan State University College of Human Medicine, 16001 W Nine Mile Rd, Southfield, MI 48075 USA; 6 Department of Biomedical Engineering, Wayne State University, 818 W Hancock St, Detroit, MI 48202 USA; 7 Flux Neuroscience, LLC, Troy, ID 83871 USA; 8 Department of Chemical and Biological Engineering, University of Idaho, 875 Perimeter Dr, Moscow, ID 4260 USA; 9 Department of Chemical Engineering and Materials Science, Wayne State University, 6135 Woodward Avenue, Rm 3120, Detroit, MI 48202 USA

**Correction: Fluids Barriers CNS (2024) 21:108**

10.1186/s12987-026-00786-6

In this article, an error was identified in the **Computational Modeling Methods** section. In the sentence describing the placement of the rake for flow capture, **“Segment 8”** was incorrectly stated and should be **“Segment 4.”**

The corrected sentence is as follows:

“The rake was placed to capture flow from the catheter tip, across the drainage-hole segments, and into Segment 4 to compare varying flow speeds (Supplemental Fig. 5–6).”

The original article has been updated.

