# Supplementary material for: Role of cerebroventricular size and surgical placement in modulating catheter flow distribution
Source: Fluids Barriers CNS. 2026 Mar 17;23:51. doi: 10.1186/s12987-026-00786-6 (PMC13045027; doi:10.1186/s12987-026-00786-6)
Supplement: Supplementary file 1 — Supplementary Material 1 [file 12987_2026_786_MOESM1_ESM.docx]

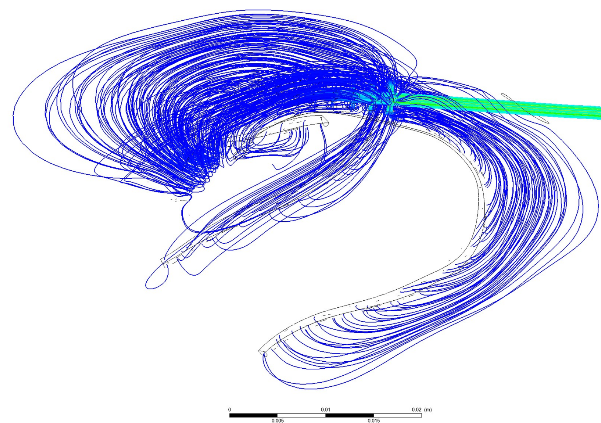

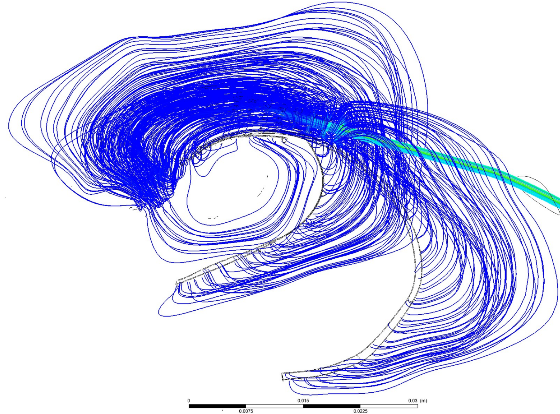

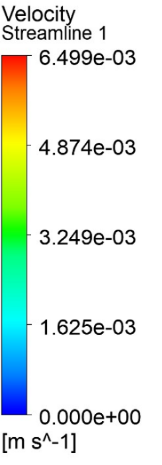

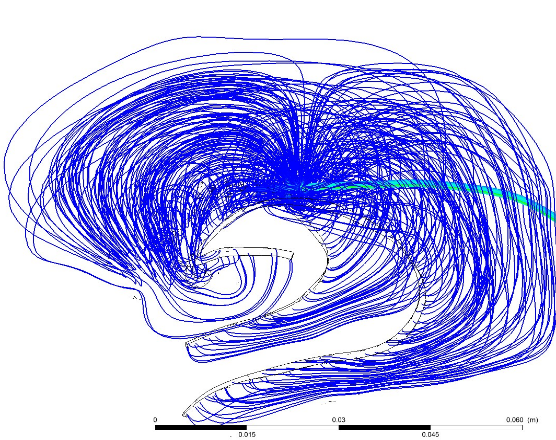


*Figure 1: Sagittal Flow Streamlines for Occipital Placement (A) Enlarged, (B) Moderate, (C) Small*


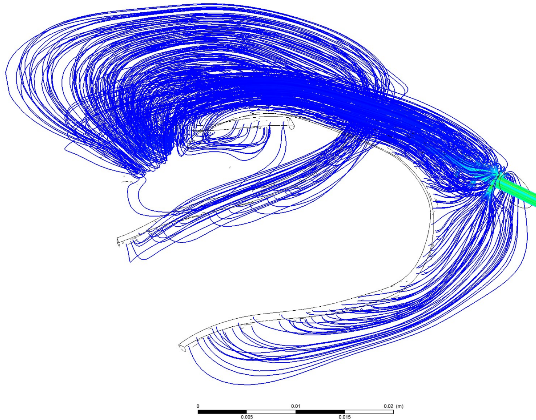

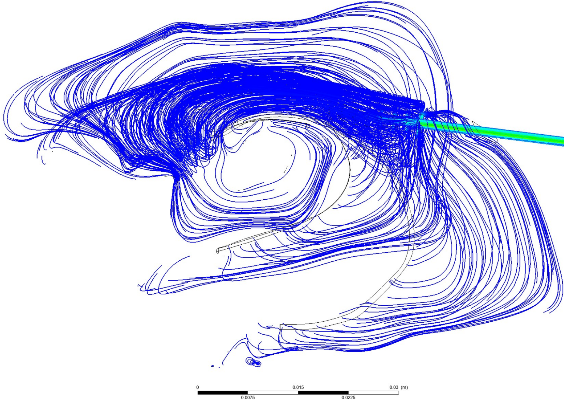

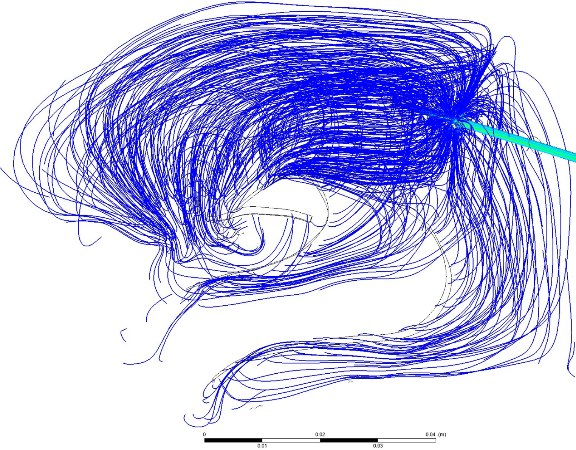


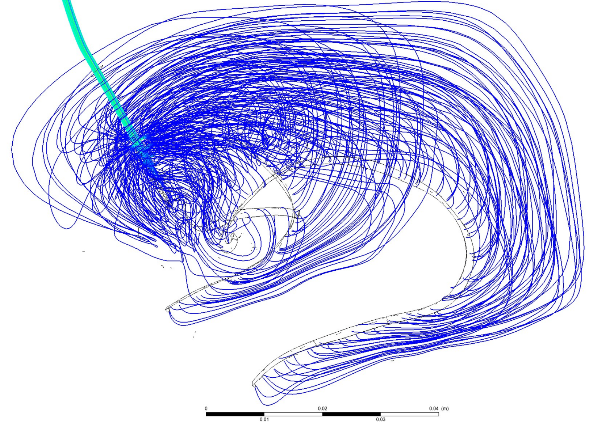

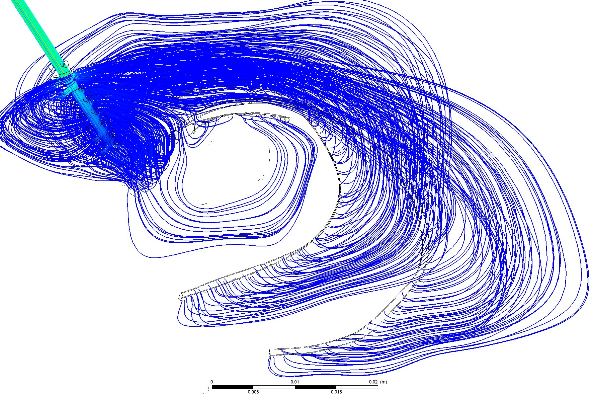
*Figure 2: Sagittal Flow Streamlines for Parietal Placement (A) Enlarged, (B) Moderate, (C) Small*


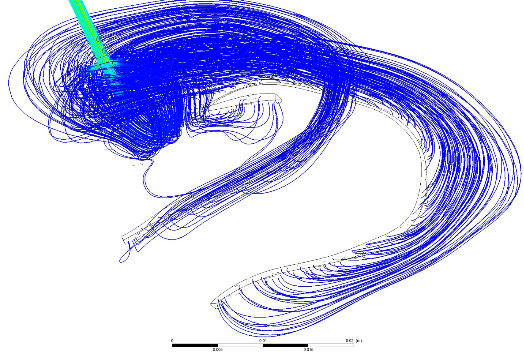


*Figure 3: Sagittal Flow Streamlines for Frontal Placement (A) Enlarged, (B) Moderate, (C) Small*


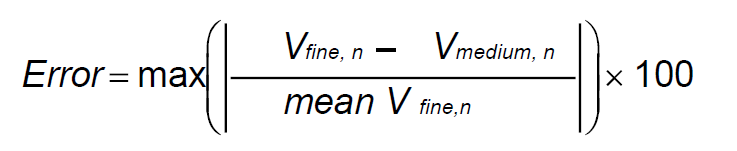


Figure 4: Relative Error Formula


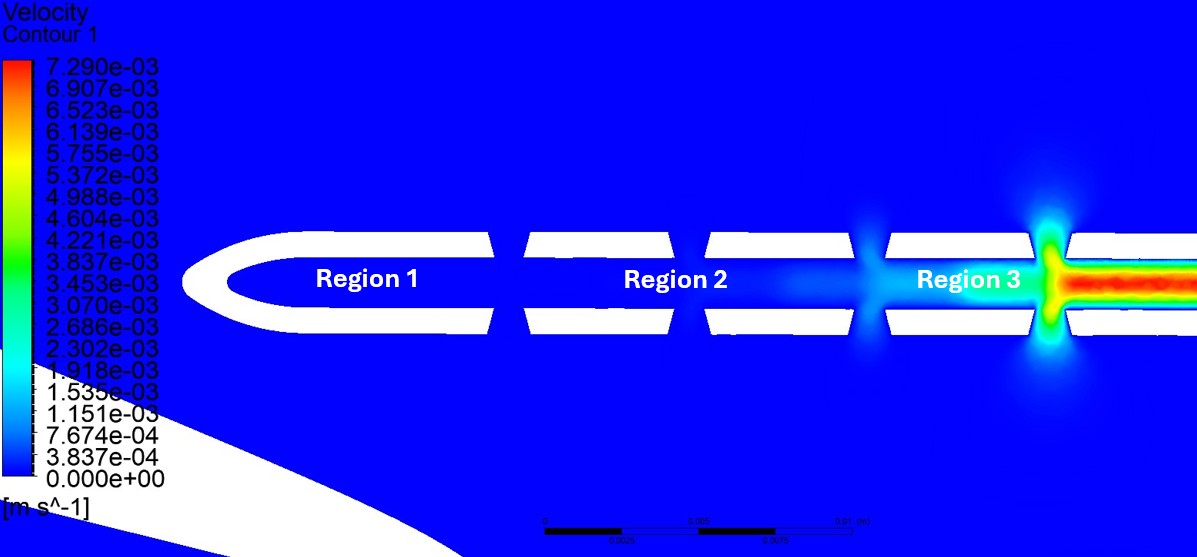


Figure 5: Velocity contours within the catheter lumen with rake sampling across three regions


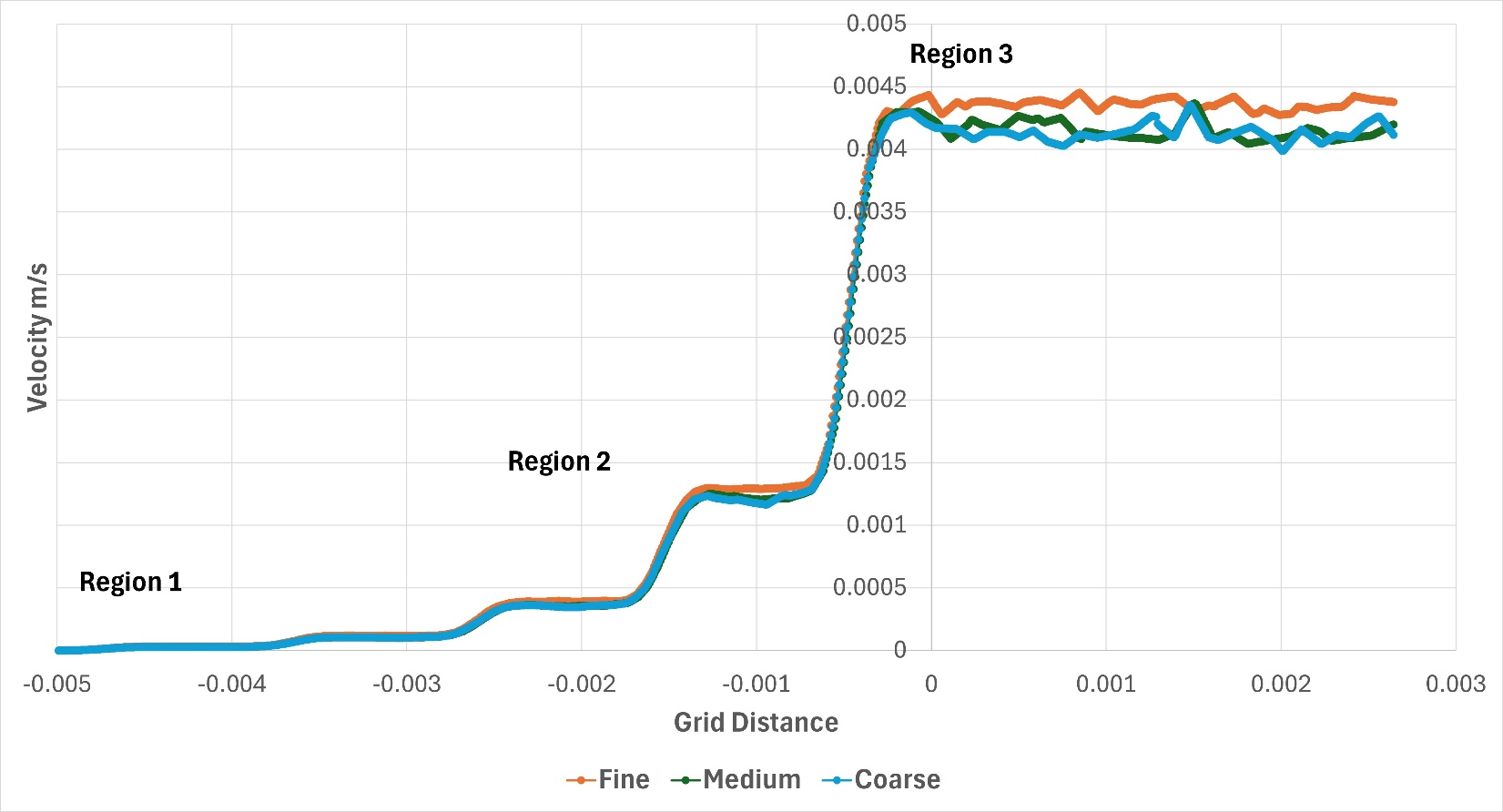


Figure 6: Line Rake through the Catheter Lumen


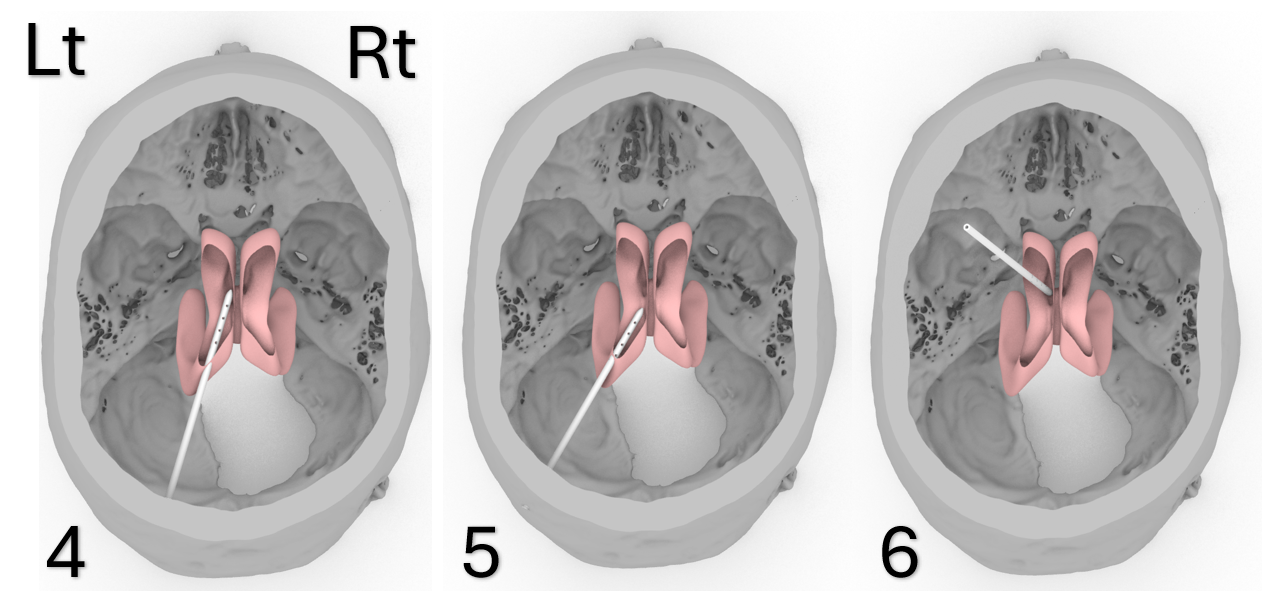


Figure 7: Moderate Ventricle Occipital Insertion (4), Parietal Insertion (5), Frontal Insertion (6)


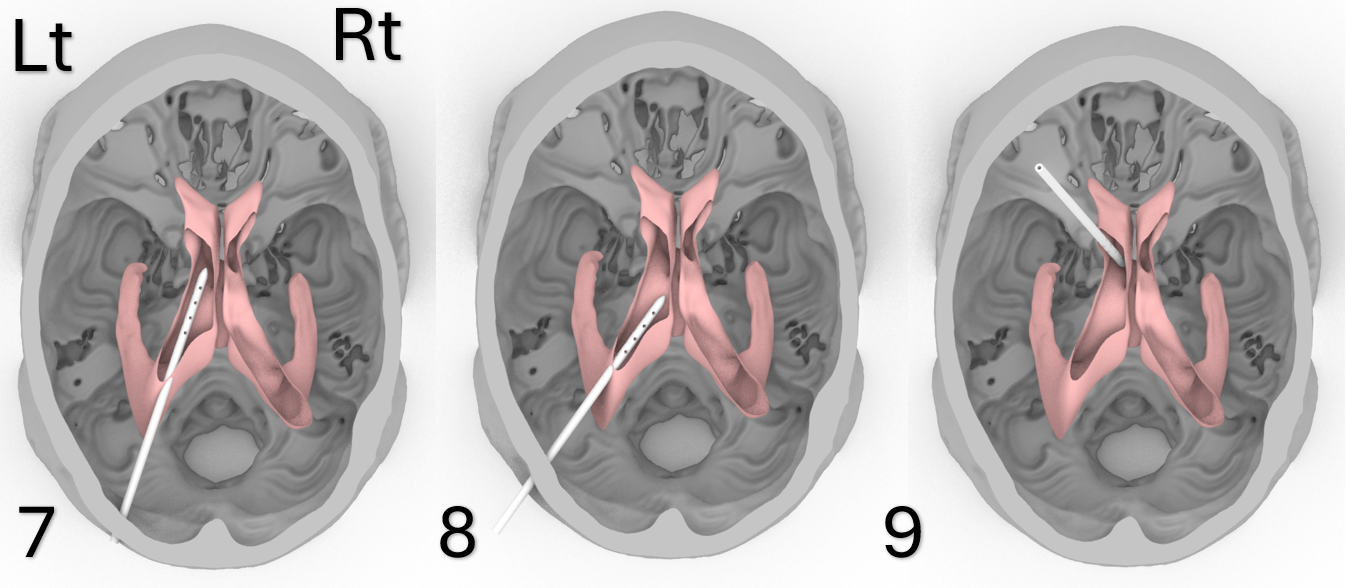


Figure 8: Small Ventricle Occipital Insertion (4), Parietal Insertion (5), Frontal Insertion (6)

**Table 1: CSF Flow Distribution Percentage**

**Occipital Placement**

| **Drainage Segments** | **Enlarged** | **Moderate** | **Small** |
| --- | --- | --- | --- |
| Segment 1 | 0.528 | 0.675 | 0.676 |
| Segment 2 | 2.5 | 2.64 | 2.67 |
| Segment 3 | 14 | 14 | 13.9 |
| Segment 4 | 81.9 | 81.6 | 81.4 |

**Frontal Placement**

| **Drainage Segments** | **Enlarged** | **Moderate** | **Small** |
| --- | --- | --- | --- |
| Segment 1 | 0.525 | 23.9 | 15.1 |
| Segment 2 | 2.5 | 75.6 | 18.7 |
| Segment 3 | 14.1 | 0.0335 | 66 |
| Segment 4 | 82.2 | 0.00973 | 0.0365 |

**Parietal Placement**

| **Drainage Segments** | **Enlarged** | **Moderate** | **Small** |
| --- | --- | --- | --- |
| Segment 1 | 0.527 | 1.91 | 0.538 |
| Segment 2 | 2.5 | 8.48 | 2.5 |
| Segment 3 | 14 | 47.9 | 14 |
| Segment 4 | 82.2 | 40.6 | 81.9 |
